# Future Biomarkers for Infection and Inflammation in Febrile Children

**DOI:** 10.3389/fimmu.2021.631308

**Published:** 2021-05-17

**Authors:** Judith Zandstra, Ilse Jongerius, Taco W. Kuijpers

**Affiliations:** ^1^ Division Research and Landsteiner Laboratory, Department of Immunopathology, Sanquin Blood Supply, Amsterdam University Medical Center (UMC), Amsterdam, Netherlands; ^2^ Department of Pediatric Immunology, Rheumatology and Infectious Diseases, Emma Children’s Hospital, Amsterdam UMC, Amsterdam, Netherlands; ^3^ Division Research and Landsteiner Laboratory, Department of Blood Cell Research, Sanquin Blood Supply, Amsterdam UMC, Amsterdam, Netherlands

**Keywords:** biomarker, febrile children, bacterial infection, viral infection, inflammation

## Abstract

Febrile patients, suffering from an infection, inflammatory disease or autoimmunity may present with similar or overlapping clinical symptoms, which makes early diagnosis difficult. Therefore, biomarkers are needed to help physicians form a correct diagnosis and initiate the right treatment to improve patient outcomes following first presentation or admittance to hospital. Here, we review the landscape of novel biomarkers and approaches of biomarker discovery. We first discuss the use of current plasma parameters and whole blood biomarkers, including results obtained by RNA profiling and mass spectrometry, to discriminate between bacterial and viral infections. Next we expand upon the use of biomarkers to distinguish between infectious and non-infectious disease. Finally, we discuss the strengths as well as the potential pitfalls of current developments. We conclude that the use of combination tests, using either protein markers or transcriptomic analysis, have advanced considerably and should be further explored to improve current diagnostics regarding febrile infections and inflammation. If proven effective when combined, these biomarker signatures will greatly accelerate early and tailored treatment decisions.

## Introduction

Fever is a common symptom in children and adults experiencing a wide variety of conditions. Fever in children signifies systemic inflammation, typically in response to a bacterial, viral, and sometimes parasitic infection, or less commonly, a non-infectious etiology (1).

Additional causes of fever must be considered for adults, malignancies in particular (2). To discriminate between a broad range of infectious and non-infectious febrile diseases, physicians must rely on longstanding experience, interviewing skills and thorough physical examination, as well as the right diagnostic tools such as microbiological tests and imaging (3). In many cases the exact trigger for the febrile condition is not found immediately at the first consultation or admittance to the hospital (4) and antibiotics, sometimes combined with antiviral medication, are started (5). For optimal treatment of disease, rapid diagnosis is of the essence and early diagnostic biomarkers that can reliably indicate the cause of fever within hours by rapid testing, are still lacking.

As mentioned, antibiotics are often prescribed whilst a physician is uncertain whether a bacterial or viral infection is present. This is particular true for children with little capacity to express themselves and in whom disease progression may take place more rapidly than in adults. This extensive use of antimicrobials comes with a price: inducing antimicrobial resistance which is increasing worldwide (5–8). The first important goal for using biomarkers in diagnostics is to discriminate between bacterial and viral infections in febrile patients, so limiting antibiotic use. Additional to bacterial and viral infection, parasitic infections cause a tremendous burden of disease in both the tropics and subtropics. Malaria, is not included here because of the many tests available to rapidly diagnose this infection (9), despite being the only parasite infestation that does cause fever in the acute stage of infection (10)

Beyond the distinction between bacterial versus viral disease, fever can be part of the early presentation of many autoinflammatory or autoimmune diseases. These are rare, more difficult to diagnose and is often mistaken for an infectious disorder. In the most severe presentations of an infection, we observe that almost all septic patients also suffer from systemic inflammation. However, not all patients with a systemic inflammatory syndrome are septic or suffer from a systemic viral disease. Autoinflammatory diseases are characterized by prolonged or recurrent febrile episodes, with a predominant dysregulation of the innate immune system and absence of autoantibody production (11), whereas autoimmune diseases are mostly driven by the adaptive immunity with involvement of dysregulated T- and B-cell responses (12). In such conditions, fever is a hallmark of disease and can be easily misjudged for, or even triggered by, an infection (13, 14). Correct treatment varies per disease, but involves immunosuppressive drugs or biologic therapies targeting specific cellular mechanisms, immune cells or cytokines of the immune system (15). Therefore, the second important goal of biomarkers is to distinguish between infectious and non-infectious disease.

As described, it is difficult to distinguish between infection, inflammation and autoimmunity. Biomarkers can, in combination with the presenting symptoms of the patient, help physicians to consider a proper diagnosis and treatment. In addition, specific biomarkers might also make it possible to bypass laborious microbiological tests and avoid invasive diagnostic procedures (such as bronchoalveolar lavage, biopsies or surgical procedures), while starting effective therapies for non-infectious inflammatory or autoimmune disease more promptly upon its onset (16).

### CRP and PCT as Current Biomarkers in the Clinic

**Figure 1:**
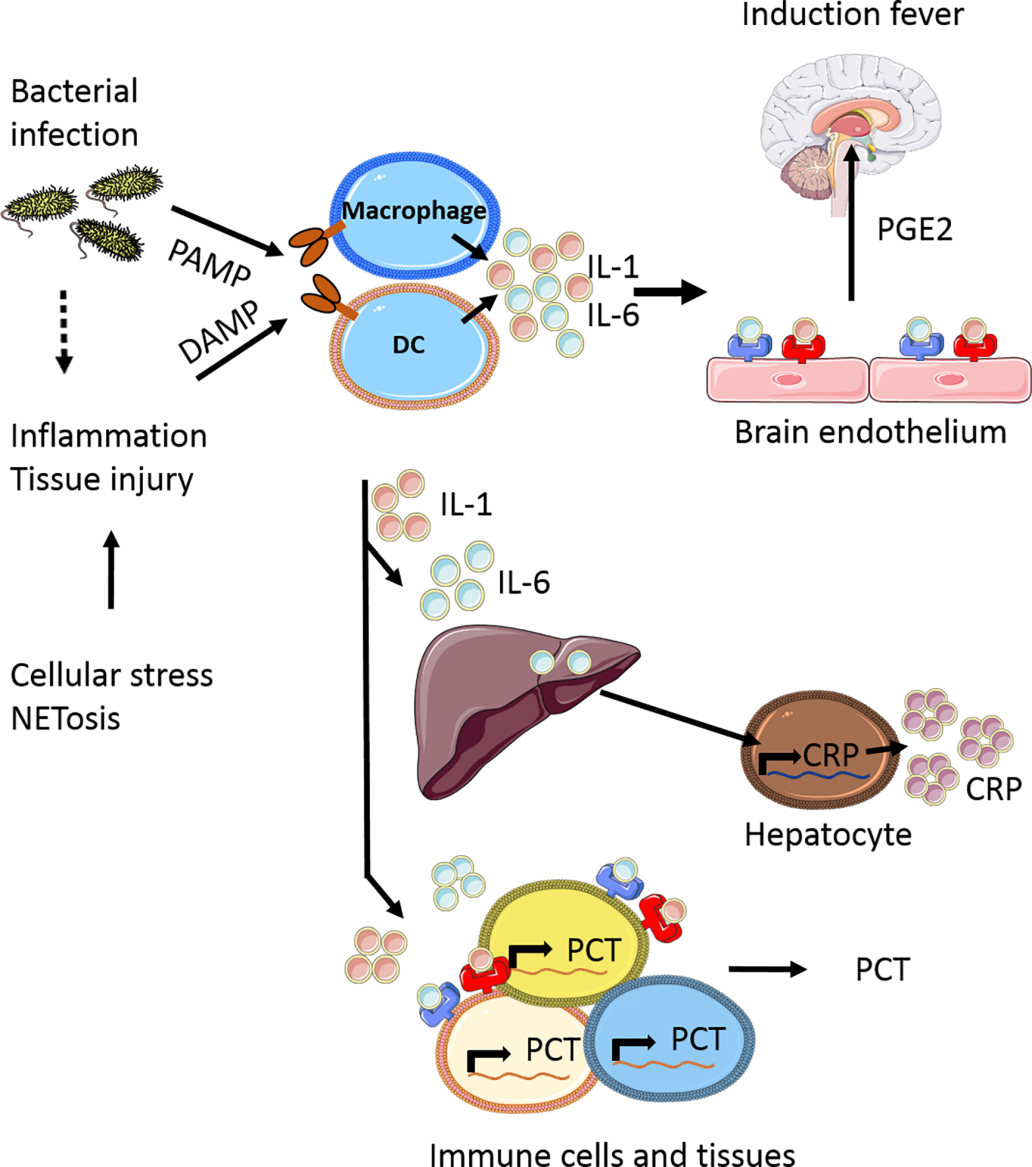
Simplified scheme of the induction of fever and release acute phase response proteins. Upon bacterial infection pathogen-associated molecular patterns (PAMPs) are released into the circulations which can be recognized by various cells, including macrophages and dendritic cells (DC). At the same time inflammation can occur, either as result of the infection or by tissue injury due to NETosis, leading to a release of damage-associated molecular patterns (DAMPs). In response to DAMPs and PAMPs recognition by several pattern recognition receptors (PPRs) receptor families, being ultimately involved in the production of pro-inflammatory cytokines, including IL-1 and IL-6. These cytokines are released and can bind to specialized regions of brain endothelium, which in response produces prostaglandin E2 (PGE2) as one of the major mediators to induce fever (17). In addition to fever, IL-1 and IL-6 also induce C-reactive protein (CRP) production in the liver and procalcitonin (PCT) production by various immune cells (macrophages, monocytes) and tissues (liver, spleen, lung), which are the most common biomarkers currently measured in the clinical setting of febrile disease, as part of the acute phase response. This figure is made using Sevier Medical Art.

Upon infection, in particular bacterial infection, pro-inflammatory cytokines such as IL-1 and IL-6 are released into the circulation in response to pathogen-associated molecular patterns (PAMPs) (17). Additionally, during inflammation and tissue injury, damage-associated molecular patterns (DAMPs) can be released from injured tissue cells, activating immune cells by Toll Like Receptor (TLR) signaling to generate and release IL-1 and IL-6. Both these cytokines have been shown to induce fever by binding respectively to the IL-1 receptor (18) and IL-6 receptor (19) on endothelial cells in the brain, especially in the hypothalamus. IL-1 induces ubiquitous IL-6 synthesis in many cells including hepatocytes and macrophages in the liver (i.e. Kupffer cells), which in turn stimulates CRP production in the liver as part of the acute phase response (**Figure 1**). CRP regulates important inflammatory processes, such as activating the complement system, stimulating apoptosis, inducing release of proinflammatory cytokines (20). In the case of uncommon primary genetic defects in these signaling cascades, or by blocking either IL-1 or IL-6 signaling, fever is largely prevented and no CRP is released (21, 22).

IL-1 and IL-6 have been studied as biomarkers for bacterial infection but fail to show sufficient specificity and sensitivity in both children (16, 23) and adults (24), most likely because of the extremely short half-life of both cytokines (25). In contrast, CRP is more stable with a half-life of 18-20 hours and has become the most commonly used biomarker for bacterial infection and inflammation and is routinely measured in the clinic nowadays for both children and adults. Next to CRP, procalcitonin (PCT) is often used as biomarker for bacterial infections and inflammation. Under normal conditions, PCT is produced by neuroendocrine C cells of the thyroid as the 116-amino acid precursor of calcitonin. During bacterial infection, PCT is upregulated and consequently expressed in all cells of the body (26), but mainly by hepatocytes (27), leading to the release of elevated amounts of PCT in the circulation (26). PCT levels rise quickly after infection, reaching a peak between 12-24 hours. This is earlier than CRP, which peaks after 2-3 days (20). With a half-life of 24-30 hours PCT can stimulate the release of pro-inflammatory cytokines, amplifying inflammatory responses (28). PCT is a better discriminator between viral or bacterial infections compared to CRP (29). This is due to the fact that many inflammatory cytokines, including IL-1 and IL-6, contribute to the up-regulation of PCT. An exception is interferon-gamma (IFN-γ) which reduces PCT expression, therefore resulting in the lower concentrations of PCT found in viral infections (30). In addition, it has been suggested that PCT is particularly useful in detecting Gram-negative infections, with better discrimination compared to CRP (31, 32). Gram-negative and Gram-positive bacteria activate mostly TLR-4 or TLR-2, respectively (33), leading to the activation of different signaling pathways and distinct cytokine profiles (34).

Apart from CRP, PCT is routinely measured in some but not all clinics. Whereas CRP is broadly used as diagnostic biomarkers for bacterial infections, PCT is predominantly applied as a diagnostic biomarker in neonatal sepsis (35). Under circumstances where viral infection is less likely both CRP and PCT are good indicators for the need to treat with antibiotics, such as in febrile neonates and very young infants when disease is often caused by *Streptococcus agalactiae* or *Escherichia coli* infection postpartum (36). However, the absence of standardized algorithms for the use of PCT – also in the field of neonatology – makes interpretation difficult (37, 38). In addition, also in some viral infections CRP and PCT can be increased, as observed in enteroviral or respiratory syncytial virus infections, as well as in severe SARS-CoV-2 infections with concomitant inflammation (39–42). Irrespective of age, both CRP and PCT concentrations rise in response to both bacterial infection and non-infectious inflammation (43–45). Although potentially useful for disease-monitoring purposes (46–48), these two biomarkers may be sensitive but not specific to correctly discriminate early on between the type of infection or inflammation in a febrile patient.

### Potential New Biomarkers for Infection

Several studies have tried to identify other biomarkers that are more suitable to determine bacterial or viral infection, as extensively reviewed for studies until 2015 (49). Here, we discuss the most promising candidates including the latest data. A summary of this data, including study details, is displayed in **Supplementary Table 1**.

One of these markers is soluble CD14, also known as presepsin. CD14 is a receptor on monocytes and macrophages that recognizes different surface structures on Gram-positive and Gram-negative bacteria, including lipoteichoic acid and proteoglycans or lipopolysaccharides, respectively. After binding bacterial antigens, CD14 is cleaved and released as presepsin (50). As a biomarker, presepsin has a higher sensitivity but a lower specificity than PCT or CRP in diagnosing sepsis in children (51). In adults PCT and presepsin have similar diagnostic accuracy (52). In another study it was shown that presepsin alone had a high sensitivity and specificity to diagnose neonatal sepsis, which did not change in combination with CRP and/or PCT (53). Irrespective of age, pancreatic stone protein (PSP) which is secreted by the pancreas in response to stress during systemic infection, has been studied in both infants and adults to predict sepsis (54) and was developed into a point-of-care test by Abionic. However, proper validation is needed because PSP levels are elevated in various other diseases as well (55–57).

Upon viral infection, cells start producing interferons (IFNs) to inhibit viral replication as their first anti-viral response (58). Although protein assays have been developed for measuring interferon levels at extremely low (i.e. attomolar) concentrations (59), these tests are experimental and routine detection remains challenging. Hence, surrogate proteins induced by interferon are also investigated. Expression of the two Myxovirus resistance genes in humans are strictly controlled by IFNs, acting as a cytoplasmic GTPase that has activity against a wide range of viruses. In humans, MX1 and MX2 (also known as MxA and MxB respectively) are, in contrast to many other interferon-induced proteins, strictly induced by IFN and found to be increased in the plasma of patients suffering from viral infections (60, 61). MX1 is induced 1-2 hours after infection with a half-life of more than 2 days. Although seen as an interesting biomarker to indicate viral infection, its sensitivity questionable. A commercially available combination immunoassay for CRP and MX1, called FebriDx, was unable to discriminate between viral and bacterial infections in a prospective cohort of upper respiratory infections (62). To date, the test has not been extensively studied in other infection cohorts.

To date, further studies are being conducted to combine plasma biomarkers to discriminate between bacterial from viral infection, of which a selection of promising studies is shown in **Table 1**. For example, soluble TNF-related apoptosis-inducing ligand (TRAIL), IFN-γ-induced protein-10 (IP-10, CXCL10) and CRP were used in combination to distinguish between bacterial and viral infection in children and adults (63, 65). In contrast to CRP, the biomarkers TRAIL and IP-10 showed increased protein levels in viral infections (63–65, 70). Known as the ImmunoXpert test, the assay has the potential to be developed into a ready-to-use assay to prevent inappropriate antibiotic use in patients with infections, but remains to be confirmed in other cohorts to validate its performance and accuracy (63).

**Table 1:** Characteristics combined diagnostics markers.

	Disease	Biomarker	Patient group	Number of patients	AUC (95% CI)	Sensitivity (95% CI)	Specificity (95% CI)
Oved 2015 (63)	Bacterial infection vs viral infection	CRP, TRAIL, IP-10	Adults and pediatrics	Patients combined: 653	0.94 (0.92-0.96)	0.87 (0.83, 0.91)	0.90 (0.86, 0.93)
Ashkenazi 2018 (64)	Bacterial infection vs viral infection	CRP, TRAIL, IP-10	Adults and pediatrics	Adults: 111 Pediatrics: 203	0.94 (0.91-0.97)	0.94 (0.89-0.98)	0.94 (0.91-0.98)
van Houten 2017 (65)	Bacterial infection vs viral infection	CRP, TRAIL, IP-10	Pediatrics	577	0.90 (0.86-0.95)	0.87 (0.76-0.93)	0.91 0.88-0.94)
Self 2017 (62)	Bacterial infection vs viral infection	CRP, Mx1	Pediatrics	205	Not reported	0.60 (0.16-0.95)	1 (94-1)
Ruan 2018 (53)	Sepsis	CRP, PCT	Neonates	2661, meta-analysis	0.96 (0.93-0.97)	0.91 (0.84-0.95)	0.89 (0.81-0.93)
Song 2019 (66)	Sepsis	CRP, CD64	Neonates	1114, meta-analysis	0.96 (0.94-0.97)	0.95 (0.86-0.98)	0.86 (0.74-0.93)
Nuutila 2013 (67)	Bacterial infection vs viral infection	CD32, CD35, CD88, MHC class I	Adults	205	Not reported	0.91	0.92
Tremoulet 2015 (68)	KD	8 panel	Pediatrics	102	0.81-0.96	Not reported	Not reported
Zandstra 2020 (69)	KD vs infection	CRP, S100A8/A9, HNE	Pediatrics	404	0.84 (0.80-0.88)	0.74	0.83

Detailed information of the combined diagnostics biomarkers; including disease; used biomarker and patient group. If reported the area under the curve (AUC); sensitivity and specificity; all with 95% confidence interval (CI); are stated. CRP, C-reactive protein; HNE, human neutrophil elastase; IP-10, Interferon gamma-induced protein 10; MHC class I, major histocompatibility complex class I; Mx1, Myxovirus resistance protein 1; PCT, Procalcitonin; TRAIL, TNF-related apoptosis-inducing ligand.

In addition to soluble plasma markers of cell-derived surface proteins, cellular expression of surface proteins themselves have been investigated as biomarkers linked to cell activation during infectious disease (49). Most studies include surface proteins on neutrophils activated during bacterial infections, or monocytes which can increase in number during viral infections. An accurate diagnosis of bacterial or viral infection within 1 hour using flow cytometry was possible using a set of surface proteins on neutrophils and monocytes, including Fcγ receptor I (FcγRI/CD64), FcγRII/CD32, complement receptor 1 (CR1/CD35), HLA-class-I, and the receptor for complement-derived anaphylatoxin C5a (C5aR/CD88) (67). CD32, CD35 and CD88 all have a higher expression on both neutrophils and monocytes in bacterial infections compared to viral infections, whereas HLA-class-I and CD169 on monocytes were increased in viral infections rather than bacterial infections (71, 72). These studies only included adults, no data on the pediatric population is available.

In neonatal sepsis, other neutrophil membrane bound proteins such as CD11b and CD64 were studied (73, 74). The meta-analysis for CD11b showed a very wide range in sensitivity and specificity between 0.56-1.00. A comparable range between sensitivity and specificity was observed for CD64. This variation might be due to the different cut-off values used in the studies, making it difficult to compare the overall sensitivity and specificity. Further studies are needed before implementation of such cellular assays as routine diagnostic test in the clinic can be considered. We also need to take into account that cell-based assays will require robust logistics and expertise making high-throughput application and interpretation much more demanding than a straight-forward ELISA system that can be easily repeated on the same sample when failed.

To conclude, a single protein marker that can distinguish bacterial from viral infections has not yet been identified. Promising results are shown by combining different markers. At the moment, large-scale studies using a heterogeneous research population are needed to bridge the gap to transform research findings into clinical diagnostics.

### Biomarkers in Inflammatory Diseases

Separating infectious from non-infectious disease is key, as the diagnosis and treatment of non-infectious disease is totally different from that of infections (11, 75). As discussed, detection of most cytokines is challenging because of their very short half-life. One notable exception is the prolonged presence of strongly increased levels of circulating IL-18 in systemic non-infectious inflammatory disorders. Initially described as an IFN-γ inducing factor, IL-18 is involved in Th1 response, NK cell activation, and results in macrophage activation. IL-18, a member of the IL-1 family, is processed by caspase-1 to an active mature form that binds to its specific receptor (76). IL-18 can be determined in plasma as a biomarker because it is protected from clearance by associating with a plasma protein, IL-18 binding protein (IL-18BP), that can also be therapeutically applied to neutralize the biological activity of IL-18 (77).

IL-18 and IL-1 are involved in classic autoinflammatory diseases including Familial Mediterranean Fever, cryopyrinopathies, and hyperimmunoglobulin D Syndrome (11), in which the enzyme protein complex known as the inflammasome that processes the pro-forms of IL-18 and IL-1 is dysregulated by genetic mutations. These diseases are often considered because of the recurrence of non-infectious febrile episodes, which often start during early childhood. Biomarker screening that includes IL-18 may help to confirm severe autoinflammation. These autoinflammatory disorders encompass non-inherited systemic onset juvenile idiopathic arthritis (soJIA) (78, 79), adult-onset systemic arthritis (Still’s disease) (80), or hemophagocytic syndrome (81, 82), the latter being also known as Macrophage Activation Syndrome (MAS) secondary to rheumatic diseases like soJIA and Still’s disease. Although the exact mechanisms involved in the development of MAS are not fully understood, increasing evidence demonstrate the role of IL-18 in upregulating the production of IFN-γ (83). In such severe autoinflammatory disorders, circulating IL-18 is readily measured at high levels while IL-1 cannot easily be detected even though it is actively involved in the disease process. These severe febrile disorders are preferably treated as early as possible with the recombinant IL-1 Receptor antagonists (84) or, still experimentally, IL-18BP (85), so avoiding the administration of antimicrobials. Classic inflammasome defects are not associated with MAS (11), which seems to correspond with much lower IL-18 levels, suggesting that high levels of IL-18 may be the necessary link between inflammasome hyperactivity and secondary HLH/MAS (86). Further to this, IFN-γ-induced biomarkers like CXCL10 (79) or CXCL9 (87) may become important biomarkers to include in multiplexed diagnostic tests.

Numerous other biomarkers are studied for diagnosis and monitoring of autoinflammatory diseases (88, 89). S100A8/A9 and S100A12 are good biomarkers for diagnostic purposes, monitoring disease activity, predicting treatment response, or risk for relapse in such non-infectious febrile diseases (90), including soJIA, adult Still’s disease and the above mentioned classic fever syndromes. S100A8/A9 protein dimers are also known as the MRP8/14 heterodimer or more commonly calprotectin. This biomarker has already been used for more than a decade as a diagnostic test in fecal samples from patients with inflammatory bowel disease (91). S100A8/A9 as well as the homodimer S100A12 proteins are released from activated innate cells, mostly neutrophils, and are of direct pathophysiologic interest (92). As extensively reviewed (92), S100 proteins have direct inflammation-propagating activity once released from neutrophils (or monocytes) as local or systemic activating signals of the innate cells by binding to the TLR4 and the ‘Receptor for Advanced Glycation End products’ (RAGE), respectively. TLR4 is a multiligand receptor for PAMPs of various bacterial pathogens as well as DAMPs, while RAGE is a multiligand member of the immunoglobulin superfamily of cell surface molecules, interacts with distinct molecules implicated in homeostasis and inflammation. Both are abundantly expressed on immune cells, endothelial cells and other extravascular tissue cells, which may contribute to initiate and propagate systemic disease and multiorgan failure during invasive infections or inflammation.

Autoimmune diseases are mostly driven by the adaptive immune system, with the predominant involvement of autoreactive T- and B-cell responses (12). There are at least 80 types of autoimmune diseases (12). However, a strict separation between autoimmunity and autoinflammation may sometimes be very difficult. Autoimmune diseases are to some extent overlapping disorders because of concomitant inflammation in which a role for innate immune cells is more prominent. For example, while systemic lupus erythematosus (SLE) is an autoantibody and immune complex disease by nature, most of its organ manifestations are in fact inflammatory. This may also apply to IBD and autoantibody-associated vasculitis. Hence, proinflammatory cytokines are not only involved in autoinflammatory disease but also autoimmune diseases, as shown by strong upregulation of IL-1 and IL-18 in active SLE (93).

One example of vasculitis that usually presents as a febrile disease in childhood is Kawasaki disease (KD). Apart from the high and persistent fever, the clinical symptoms in KD mimic both bacterial and viral disease presenting with lymphadenopathy, skin rash, red eyes, red tongue and/or red hands and feet, in the presence of a raised CRP. KD is often misdiagnosed for streptococcal infection or a measles-like viral disease. No blood test for KD is available to date and the diagnosis is based on clinical criteria (94). Treatment with a single high-dose intravenous immunoglobulin (IVIG) helps to quickly recover from and protects against the development of coronary artery aneurysms. This complication is why the disease is feared and should be recognized swiftly to administer the IVIG as soon as possible. To discriminate KD from infectious disease, many different biomarkers have been studied over the years but lack good specificity and sensitivity, with many studies lacking proper control groups (95). Recently, we showed that a combination of three biomarkers; CRP, neutrophil elastase and S100A8/A9, reached high sensitivity and specificity in KD to efficiently differentiate KD from infectious disease in febrile controls (69). To validate these findings, a new study comparing children with KD and other types of vasculitis or inflammatory syndromes is necessary before developing a point-of-care test to implement this combination of biomarkers in the clinic.

In summary, when febrile episodes mimic infections tests should not only separate bacterial from viral disease, but also discriminate infectious from inflammatory disorders.

### Transcriptomics

Recent studies show that highly sensitive and specific biomarkers can be found using RNA transcript analysis. RNA transcriptional activity changes during the course of disease leading to different transcript levels, new transcripts or splice variants when compared to the healthy state or among different diseases (96). As discussed, there is a serious advantage for the use of multiple protein markers at the same time to distinguish the cause of fever. In this respect, unbiased transcriptome analysis may account for thousands of transcripts, allowing an even more in-depth refinement of the possible cause of a given febrile disease. In agreement with previous results, many gene transcripts that are upregulated are related to interferon signaling in viral disease (97) while integrin-related gene transcripts are more prominent in bacterial disease (98) (**Table 2**). The overwhelming number of transcripts obtained by transcriptomics need to be reduced to make a potentially rapid test result available in the near future. This has led to a 2-transcript host RNA signature of combining *FAM89A* and *IFI44L* to discriminate between bacterial or viral infection in children (101), which has been modified to the combination of *EMR1-ADGRE1* and *IFI44L* when the signature was further developed into a point-of-care test (112). Using these RNA signatures it was shown that while >90% of patients present in the group of unknown infections received antibiotic, only 46% of this group had a bacterial infection (101). This study again shows the excessive use of antibiotics in infections and the need to improve diagnostics for febrile children.

**Table 2:** Characteristics transcriptomic biomarkers.

	Disease	Transcript	Patient group	Number of patients	AUC (95% CI)	Sensitivity (95% CI)	Specificity (95% CI)
Ramilo 2007 (99)	Bacterial infection vs viral infection	35 genes	Pediatrics	131	No AUC reported. Prediction accuracy of 95% in discriminating bacterial or viral infection		
Xinran 2013 (100)	Bacterial infection vs viral infection	1581 genes, and selection of 18-33 genes	Pediatrics	30	Prediction accuracy between 77% and 90%.		
Herberg 2016 (101)	Bacterial infection vs viral infection	*FAM89A, IFI44L*	Pediatrics	370	0.97 (0.91-1)	1 (0.85-1)	0.96 (0.89-1)
Kaforou 2017 (102)	Bacterial infection vs viral infection	*FAM89A, IFI44L*	Infants <60 days	279	0.96 (0.93-0.98)	0.89 (0.80-0.95)	0.94 (0.87-0.97)
Andres-Terre 2015 (103)	Influenza vs bacterial infections	*CD38, HERC5, HERC6, IF16, IFIH1, LGLS3BP, LY6E, MX1, PARP12, RTP4, XAF1, ZBP1*	Pediatrics	2939, meta-analysis	0.94 (0.98-0.9)	Not reported	Not reported
Heinonen 2016 (104)	Rhinovirus	393 genes	Pediatrics	151	AUC not reported. Gene transcript highly upregulated in patients with symptomatic rhinovirus infection compared to asymptomatic patients and controls		
Mayhew 2020 (105)	Bacterial vs viral or inflammation	29 genes	Adults	1015, meta-analysis	0.92 (0.83-0.99)	Not reported	Not reported
	Viral vs bacterial or inflammation				0.91 (0.82-0.98)		
Mahajan 2016 (106)	With and without bacterial infection	66 genes	Infants <60 days	279	Not reported	0.87 (0.73-0.95)	0.89 (0.81-0.93)
	Bacterial infection vs serious bacterial infection	*BATF, MSRA, ALOX5AP, PAD14, RA27A, FCAR, MGAM, HNRNPA3P1, MMP9, HSH2D*				0.94 (0.70-1)	0.95 (0.88-0.98)
Sampson 2017 (107)	Viral vs non-viral systemic inflammation	*ISG15, IL16, OASL, ADGRE5*	Adults and pediatrics	1754, meta-analysis	AUC not reported. The four-gene signature is statistically significant in 13 validation datasets to discriminate between viral and non-viral inflammatory conditions		
Blohmke 2019 (108)	Enteric fever vs other febrile disease	*STAT1, SLAMF8, PSME2, WARS, ALDH1A1*	Adults		0.97 (0.94-1)	0.97	0.88
Wright 2018 (109)	KD vs febrile disease	*CACNA1E, DDIAS, KLHL2, PYROXD2, SMOX, ZNF185, LINC02035, CLIC3, S100P, IFI27, HS.553068, CD163, RTN1*	Pediatrics	606	0.96 (0.93-0.99)	0.82 (0.60-0.95)	0.92 (0.84-0.97)
Wu 2019 (110)	SLE vs RA vs Sjögren’s syndrome	Long non-coding RNAlinc0597, GAS5, lnc0640, lnc5150, lnc7074	Adults	325	0.80 (0.72-0.86)	0.68	0.82
Sweeney 2017 (111)	Active tuberculosis vs latent tuberculosis	*GBP5, DUSHP3, KLF2*	Adults and pediatrics	2572, meta-analysis	0.88 (0.84-0.92)	0.80	0.86

Detailed information of the combined diagnostics transcripts, including disease, used transcripts and patient group. If reported the area under the curve (AUC), sensitivity and specificity, all with 95% confidence interval (CI), are stated.

**Figure 2:**
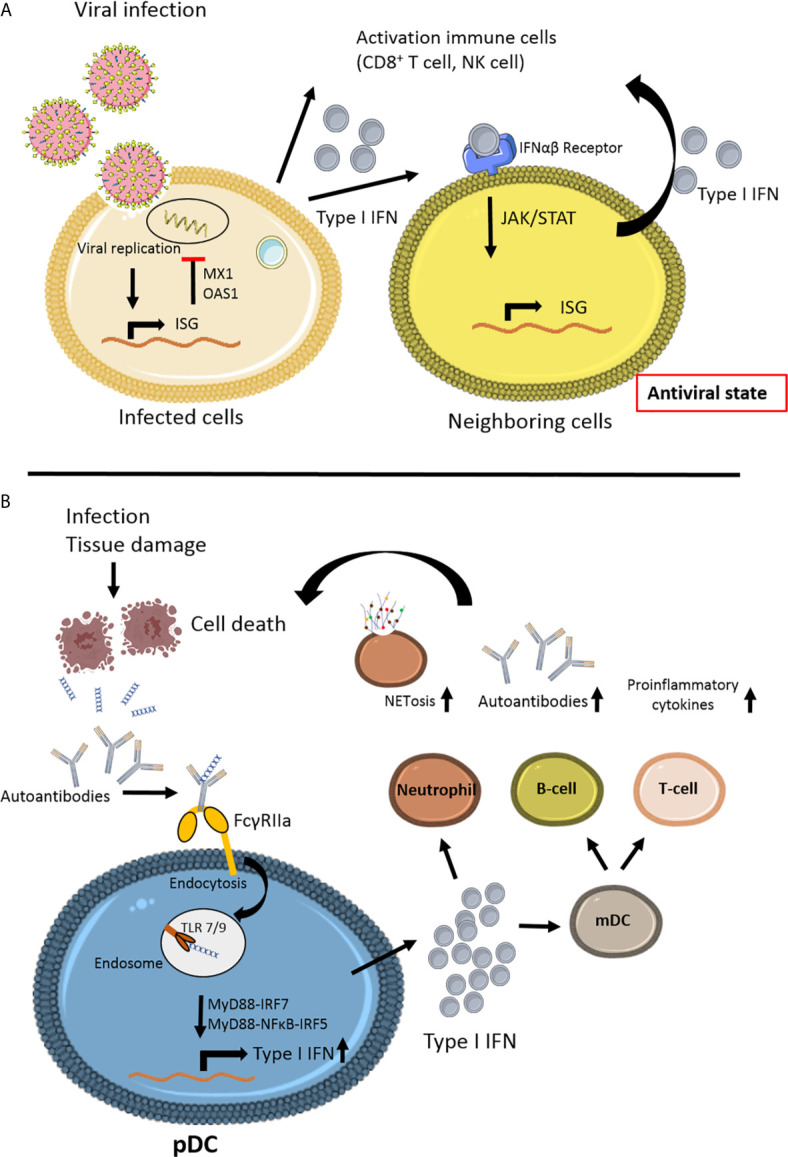
Schematic view of the different steps of interferon signaling involved in viral infection and autoimmune disease. **(A)** Viral infection induces interferon stimulated gene (ISG) stimulation (58). Antiviral proteins (MX1, OAS1) will inhibit viral replication in the cell. Secretion of Type I IFN by the infected cell will lead to paracrine signaling to the neighboring cell *via* JAK/STAT signaling, thereby stimulating ISG in non-infecting cells, leading to an antiviral state, preventing further viral infection. The anti-viral immune response is induced by activating various immune cells, especially CD8^+^ T cell survival and natural killer (NK) cell activation. **(B)** In autoimmune disease, such as Systemic Lupus Erythematosus (SLE), a variety of environmental factors can trigger the disease in genetically predisposed individuals (12). Infection or tissue damage can be an example of a trigger for autoimmune disease by the formation of autoantibody complexes with DNA released from apoptotic cells. The antibodies bind to the IgG receptor FcγRIIa and *via* endocytosis can activate endosomal Toll like receptor (TLR)7 and TLR9 (117). Due to mutations in JAK/STAT and/or interferon regulating factors IRFs the release of Type I IFN is upregulated in plasmacytoid dendritic cells (pDC). High levels of Type I IFNs can activate T- and B-cells *via* myeloid DCs, leading to more autoantibodies and proinflammatory cytokines. Type I IFN can promote cytotoxicity NK cells, phagocytosis in macrophages and NETosis in neutrophils (118, 119). This figure is made using Sevier Medical Art.

The next challenge lies in the fact that biomarkers are needed to distinguish between viral infections and inflammatory disease. Whereas the abovementioned *IFI44L* acts as viral biomarker, it is part of a more extensive set of interferon-stimulated genes (ISGs), or so-called ISG signature (58, 113). Other ISGs can be used to identify systemic autoinflammatory diseases, including SLE (114–116). The differences in viral and autoinflammatory ISGs are illustrated in **Figure 2**.

Recently, a panel of only 5 long non-coding RNAs was used to distinguish SLE patients from patients with rheumatoid arthritis and primary Sjögren’s syndrome (110). In the same way, it was shown that a 13-gene transcript signature was able to distinguish KD patients from both infectious and other inflammatory patients (109), demonstrating the power of multiplexed analysis as a leading principle. The strength of this study was the inclusion of a wide range of disorders with overlapping symptoms of KD, infectious and inflammatory patients. In addition, the proposed model was validated with an independent group of patients to strengthen their findings. Results from various studies on infectious as well as inflammatory diseases, indicate the value of using RNA transcriptome analyses for diagnostic purposes, as summarized in **Table 2**.

Although transcriptomic approaches look promising, they come with limitations. Apart from the lack of routine availability (120), another important disadvantage of transcriptomics is its cell dependence (112). Often the cellular composition may not be stable making the signatures less robust. For example, a leukopenic blood sample might have a very limited yield, most likely limiting the discriminatory results, preventing its application in oncology patients admitted with febrile neutropenia. Despite these difficulties, the implementation of transcriptomic biomarker tests into the clinic is one step closer. For the 2-transcript signature (101) a fast and highly accurate RT-qPCR was developed which efficiently distinguished between bacterial and viral infections (121), as well as a point-of-care test with the improved transcript signature (112), showing potential for rapid testing in the near future. In adults though, distinguishing bacterial disease and sepsis from viral disease or inflammation using transcriptomics was shown to be challenging if not ineffective (122–126), showing the limitations.

### Biomarkers *via* Mass Spectrometry or Multiplexed Protein Microarrays

As described for RNA signatures, recent techniques allow for further and more dynamic profiling which might enhance diagnostic power. Blood is estimated to contain over 10,000 distinct proteins (irrespective post-translationally modifications), with concentrations spanning a dynamic range up to 12 orders of magnitude (127). To date, the number of novel biomarkers that have been identified from biomarker discovery projects involving plasma profiling are still limited. Using this technique, the acute phase serum amyloid A (SAA) proteins as well as the S100 proteins have been identified as biomarker for rheumatic disease (128), confirming previous protein tests (129, 130). Moreover, in a targeted cohort of the deadly Ebola virus infection a multi/omics approach including plasma profiling showed upregulated HLA-class-I and molecules found on endothelial cells (e.g. VCAM1), macrophages (e.g. CD14 [presepsin], CD163, FCGR3A [FcgRIIIa]), and neutrophils (e.g. FCGR3B, FcgRIIIb) (131). Activation was more pronounced in fatalities, and were consistent with metabolomics and lipidomics signatures that predicted immune cell activation and sepsis-like immune responses in Ebola virus fatalities. Of interest is the increase in soluble VSIG4, a co-stimulatory molecule expressed on macrophages, which was only observed in fatalities and may thus act as a completely novel biomarker of a severe and fatal outcome. These studies show that the use of plasma profiling might result in identification of novel biomarkers that can help in the future to advance clinical management.

Although unbiased Mass Spectrometry has been used for plasma profiling and biomarker identification, there is a balance in the yield and the expectations. While fractionation at the peptide level greatly increases the depth of analysis, a major drawback has been the significant increase in the amount of time it takes to analyze a single sample. This trade-off between depth and throughput means that plasma proteomics for diagnosis and monitoring with relatively large numbers of samples results in shallow depth of coverage, whereas studies emphasizing depth of coverage typically have small sample numbers (132). For discovery of biomarkers plasma proteomics is preferred but the analysis takes days to weeks as shown for murine and human plasma samples (133, 134). Taken together, plasma profiling studies are still rare but they might potentially result in discovery of novel biomarkers to distinguish between more complex diseases or disease outcomes.

Another method for plasma protein discovery consists of the expanded aptamer-based multiplex proteins assay (SOMAscan) which can quantify more than 3000 proteins (135–139). Multiplexed detection approaches using antibodies as part of targeted protein panels such as current Luminex (140) or Olink platforms (141), are able to measure smaller number of analytes and are cheaper to use. Overall these multiplex microarray assays have been designed as discovery platforms and measure relative protein concentrations which cannot give absolute values without having internal controls and standard curves for accurate measurements within the known linear dynamic range of the aptamers used.

### The Complexity of Overlapping Disease

Often bacterial and viral infections are presented as separable disease categories, but in reality, these categories often overlay and cannot be regarded as sheer single entities. For instance, co-infections, such as Respiratory Syncytial Virus (RSV)-related bacterial bronchopneumonia in infants (142), influenza-associated *Staphylococcus aureus* pneumonia (143), or chickenpox-related streptococcal impetiginization of the skin (144), will influence diagnostic test results and interpretation (145). In fact, we may learn from the application of combination tests of biomarkers or transcripts, that co-infections of different pathogens may be more common than we previously believed as based on routine microbiology cultures and serology. The cut-off values in the interpretation of novel diagnostic biomarker approaches may be used to differentiate true co-infection from colonization, or innocent prolonged viral shedding from true co-infection, as mere examples in which interpretation of current PCR-based microbiology results are not straight-forward.

Another level of complexity consists of the infectious triggers that can initiate autoinflammatory derailment or autoimmunity, as is illustrated in the recent COVID-19 pandemic (146). Severe COVID-19 infection has a large overlap with MAS, with a systemic inflammatory reaction, concomitant high serum macrophage-derived ferritin and a life-threatening hyper-inflammation sustained by a cytokine storm which eventually leads to multi-organ failure. IL-6 is used a predictive marker of mortality (147). In the current COVID-19 era, post-viral Kawasaki-like multi-inflammatory syndrome in children (MIS-C) (148–152) or adults (MIS-A) (153) has been linked to cytokine release syndrome some weeks following an often asymptomatic SARS-CoV-2 infection. High levels of CRP as well as increased levels of cytokines including IL-6 can be detected which, as discussed earlier, are very unusual to find elevated during a viral infection (154). Some of the disease parameters [including ferritin (147)] are reminiscent of MAS, or of secondary HLH (155) in which bone marrow suppression is clearly present. Secondary forms of HLH occur more common in the context of infections (typically Epstein-Barr virus), malignancies, systemic autoimmunity or severe sepsis in otherwise genetically normal individuals (82, 156, 157). All these forms of HLH are clinically characterized by persistent fever, severe malaise, variable degrees of cytopenia and hepatosplenomegaly. Taken together, biomarkers alone can be misleading. Care should be taken in their interpretation when trying to diagnose complex clinical disease because of overlap between clinical syndromes with totally different etiologies.

## Conclusion

In this review we discuss the current landscape of biomarkers with a focus on, but not restricted to, febrile pediatric patients. Currently, apart from the common use of CRP in both adults and children, biomarkers like PCT, and IL-18 are frequently used in clinic practice. PCT is specifically used to differentiate between bacterial and viral infections in neonatal sepsis (29). IL-18 is a good biomarker for various auto-inflammatory diseases (78, 80, 81).

A rapid increase in the development of combination tests is observed. The latest developments regarding transcriptome analysis shows promising results, although proper validation is often needed to confirm diagnostic results. It was shown that a 2-transcript host RNA signature of combining *EMR1-ADGR* and *IFI44L* could discriminate between bacterial or viral infection in children (112), confirmed by the development of a point-of-care test, see **Table 2**. Further studies will need to show whether transcriptome analysis is able to distinguish between bacterial subgroups (i.e. Gram-positive and Gram-negative, atypical, or mycobacterial infections). In addition, both transcriptome analysis, and whole plasma profiling might result in the identification of novel plasma markers, expanding the repertoire of currently tested plasma markers.

To conclude, combining biomarkers, either plasma markers or transcriptomics, will improve diagnostics in febrile children similar as in the adult population. Further studies are needed to determine the exact combination of markers and techniques that together will lead to a solid diagnostic tool and its general application in infectious and non-infectious disease. We should emphasize that in this review, we have focused on the biomarker landscape for the western world. It should not be forgotten that infectious disease constitutes the major cause of childhood death worldwide. Most deaths in 2015 occurred in South Asia and sub-Saharan Africa (75% of the total burden) and underscores the need of a better understanding of the cause of infection in a febrile child (158). Therefore, there is still an urgent need to identify biomarkers that can be implemented throughout the world, adapted to local epidemiology, logistics and costs.

## Author Contributions

JZ wrote the manuscript. IJ and TK reviewed and revised the final manuscript. All authors contributed to the article and approved the submitted version.

## Funding

Supported by the European Union’s Horizon 2020 research and innovation program under Grant Agreement No. 668303.

## Conflict of Interest

The authors declare that the research was conducted in the absence of any commercial or financial relationships that could be construed as a potential conflict of interest.

## Supplementary Material

Supplementary Table 1 Single biomarkers mentioned in the manuscript. Detailed information of the single diagnostics biomarkers, including disease, used biomarker and patient group. If reported the area under the curve (AUC), sensitivity and specificity, all with 95% confidence interval (CI), are stated. IL- 6, Interleukin- 6; NT-proBNP, N-Terminal Pro-B Type Natriuretic Peptide; Mx1, Myxovirus resistance protein 1; SuPAR, soluble urokinase-type plasminogen activator receptor; PSP, pancreatic stone protein.
