# Supplementary material for: Future Biomarkers for Infection and Inflammation in Febrile Children
Source: Front Immunol. 2021 May 17;12:631308. doi: 10.3389/fimmu.2021.631308 (PMC8165271; doi:10.3389/fimmu.2021.631308)
Supplement: Supplementary Table 1 — Single biomarkers mentioned in the manuscript. Detailed information of the single diagnostics biomarkers, including disease, used biomarker and patient group. If reported the area under the curve (AUC), sensitivity and specificity, all with 95% confidence interval (CI), are stated. IL- 6, Interleukin- 6; NT-proBNP, N-Terminal Pro-B Type Natriuretic Peptide; Mx1, Myxovirus resistance protein 1; SuPAR, soluble urokinase-type plasminogen activator receptor; PSP, pancreatic stone protein. [file Table_1.docx]

**Supplementary Table 1: Single biomarkers mentioned in the manuscript**

|  | **Disease** | **Biomarker** | **Patient group** | **Number of patients** | **AUC (95% CI)** | **Sensitivity (95% CI)** | **Specificity (95% CI)** |
| --- | --- | --- | --- | --- | --- | --- | --- |
| Ruan 2018^53^ | Sepsis | Presepsin | Neonates | 2661, meta-analysis | 0.99 (0.98-1) | 0.94 (0.80-0.99) | 0.98 (0.87-1) |
| Yoon 2019^51^ | Sepsis | Presepsin | Pediatrics | 308, meta-analysis | 0.93 | 0.94 (0.74-0.99) | 0.71 (0.35-0.92) |
| Siahanidou 2014^159^ | Sepsis | SuPAR | Neonates | 47 | 0.78 (0.65-0.92) | 0.61 | 0.89 |
| Ni 2016^160^ | Bacterial infection | SuPAR | Adults | 1237, meta-analysis | 0.82 (0.79-0.85) | 0.73 (0.58-0.84) | 0.79 (0.73-0.83) |
| Schlapbach 2013^161^ | Sepsis | PSP | Neonates | 137 | 0.69 (0.59–0.80) | 0.79 | 0.62 |
| Iwase 2019^23^ | Infection vs non infection | IL-6 | Adults | 527, meta-analysis | 0.81 (0.78-0.85) | 0.73 (0.61-0.82) | 0.76 (0.61-0.87) |
| Toivonen 2015^162^ | Respiratory virus infections | Mx1 | Pediatrics | 230 | Not reported | 0.92 | 0.77 |
| Dai 2017^74^ | Sepsis | CD64 | Neonates | 2213, meta-analysis | 0.88 (0.85-0.91) | 0.80 (0.69-0.88) | 0.83 (0.71-0.90) |
| Qiu 2019^73^ | Sepsis | CD11b | Neonates | 843, meta-analysis | 0.9 | 0.82 (0.71-0.90) | 0.93 (0.62-0.99) |
| Lin 2015^163^ | KD | NT-proBNP | Pediatrics | 482, meta-analysis | 0.87 (0.83-0.89) | 0.89 (0.78-0.95) | 0.72 (0.78-0.82) |
| Degraeuwe 2015^91^ | IBD | S100A8/A9 | Pediatrics | 853, meta-analysis | 0.92 (0.89-0.94) | 0.97 (90.92-0.99) | 0.70 (0.59-0.79) |
